# Elevated tropomyosin expression is associated with epithelial–mesenchymal transition of lens epithelial cells

**DOI:** 10.1111/j.1582-4934.2012.01654.x

**Published:** 2012-12-04

**Authors:** Eri Kubo, Nailia Hasanova, Nigar Fatma, Hiroshi Sasaki, Dhirendra P Singh

**Affiliations:** a Department of Ophthalmology, Kanazawa Medical UniversityIshikawa, Japan; b Department of Ophthalmology and Visual Sciences, University of Nebraska Medical CenterOmaha, NE, USA

**Keywords:** epithelial–mesenchymal transition, tropomyosin, lens, posterior capsule opacity, anterior subcapsular fibrosis, cataract

## Abstract

Injury to lens epithelial cells (LECs) leads to epithelial–mesenchymal transition (EMT) with resultant fibrosis. The tropomyosin (Tpm) family of cytoskeleton proteins is involved in regulating and stabilizing actin microfilaments. Aberrant expression of Tpms leads to abnormal morphological changes with disintegration of epithelial integrity. The EMT of LECs has been proposed as a major cause of posterior capsule opacification (PCO) after cataract surgery. Using *in vivo* rodent PCO and human cataractous LECs, we demonstrated that the aberrant expression of rat Tpm and human Tpm1α/2β suggested their association in remodelling of the actin cytoskeleton during EMT of LECs. Expression analysis from abnormally growing LECs after lens extraction revealed elevated expression of α-smooth muscle actin (α-SMA), a marker for EMT. Importantly, these cells displayed increased expression of Tpm1α/2β following EMT/PCO formation. Expression of Tpm1α/2β was up-regulated in LECs isolated from cataractous lenses of Shumiya Cataract Rats (SCRs), compared with non-cataractous lenses. Also, LECs from human patients with nuclear cataract and anterior subcapsular fibrosis (ASF) displayed significantly increased expression of Tpm2β mRNA, suggesting that similar signalling invokes the expression of these molecules in LECs of cataractous SCR and human lenses. EMT was observed in LECs overexpressed with Tpm1α/2β, as evidenced by increased expression of α-SMA. These conditions were correlated with remodelling of actin filaments, possibly leading to EMT/PCO and ASF. The present findings may help clarify the condition of the actin cytoskeleton during morphogenetic EMT, and may contribute to development of Tpm-based inhibitors for postponing PCO and cataractogenesis.

## Introduction

Epithelial to mesenchymal transition (EMT) is the transdifferentiation of epithelial cells into mesenchymal cells. The process has been implicated as a major cause of progression of several diseases, including anterior subcapsular cataract and posterior capsular opacification (PCO) after cataract surgery [1–3]. Age-related cataracts, one of the most common chronic disorders of ageing, are the leading cause of blindness worldwide. At present, surgical intervention is the only cure [3], but unfortunately, after cataract surgery, aberrant cell growth across the lens capsule often leads to fibrosis and secondary visual loss, known as PCO, secondary cataracts or after cataracts [4].

Transforming growth factor (TGF)-β is known to be involved in producing abnormal changes by overmodulating extracellular matrix genes in lens epithelial cells (LECs), thus mimicking events in the development of human anterior subcapsular cataracts [5, 6] and PCO [7–10]. These changes include formation of fibroblastic cells accompanied by accumulation of extracellular matrix (ECM) and apoptosis [11]. Moreover, TGFβ1 promotes tissue fibrosis, transdifferentiation, myofibroblast formation and apoptosis [12, 13] by up-regulating genes encoding extracellular matrix proteins including α-smooth muscle actin (α-SMA). Other cellular abnormalities, particularly in aberrant expression of cytoskeleton and extracellular matrix proteins, are induced by overshooting of cellular signalling mediated by reactive oxygen species (ROS) [14]. ROS-induced damage to cells is related to ROS-driven abnormal signalling, which overstimulates TGF-β1-mediated signalling [14–16]. That in turn leads to overmodulation of expression of certain genes, such as α-SMA and βig-h3, changes which are implicated in induction of cataracts and PCO as well as other pathophysiological disorders of cells and tissues [15, 17, 18]. In a previous report, we showed that LECs deficient in Peroxiredoxin 6 (Prdx6) showed phenotypic changes, a characteristic of terminal cell differentiation and EMT [15]. Prdx6 provides cytoprotection against internal and external environmental stresses and plays a role in cellular signalling by detoxifying ROS and thereby controlling gene regulation [15, 19–22]. Using proteomic analysis of Prdx6-deficient (*Prdx6*^−/−^) LECs, we recently found that such cells displayed elevated expression of the cytoskeleton proteins tropomyosin (Tpm) 1α, Tpm2β and vimentin [23]. Importantly, an extrinsic supply of Prdx6 reduced Tpm2β expression. Therefore, we hypothesized that, because Tpms have been implicated in regulation of cellular activities by stabilizing ECM proteins (specifically actin microfilaments), aberrant expression of Tpm1α and Tpm2β genes is likely involved in the phenotypic alteration of *Prdx6*^−/−^ LECs in mice.

Dysregulation of proteins such as Tpm isoforms, which are involved in regulating actin dynamics, is a major indicator of cell activity modulation [24, 25]. Non-muscle Tpm isoforms are recognized and grouped as either high-molecular-weight (approximately 34–40 kD, such as Tm1, Tm2, Tm3 and Tm6) or low-molecular-weight isoforms (approximately 28–32 kD, such as Tm5) [26–28]. The balance between levels of isoforms in a given cell determines the cell's Tpm functions [24, 29–32]. However, Tpm regulation in cells under oxidative stress may be disrupted due to overactivation of TGF-β, as is the case in *Prdx6*^−/−^ cells [23, 33–37].

In earlier studies, we found that LECs from Shumiya Cataract Rat (SCR) lenses with cataract displayed reduced expression of Prdx6, and higher expression of TGFβ1 and α-SMA. The SCR is a hereditary cataractous rat strain, obtained by cross-breeding a spontaneous hypertensive rat and a Zucker fatty rat. In SCR, cataractogenesis is caused by mutations in the lanosterol synthase gene [38]. Lens opacity appears spontaneously in the perinuclear and nuclear portions in two-thirds of SCRs at 11–12 weeks of age [39]. A comparison of factors associated with progression of cataract in SCRs shows a spectrum of biochemical and phenotypic changes that is very similar to those observed in cataractogenesis induced by oxidative stress, TGFβ1 and other forms of deleterious signalling [15, 18, 19, 38]. Based on the similarities, we concluded that the SCR would be a plausible model system for studying whether the changes found in human EMT-PCO are similar to the changes in SCR cataractogenesis [23, 33, 40–42].

In the present study, using a rodent model of PCO coupled with LECs from cataractous human lenses of varying ages as model systems, we showed the involvement of Tpm during EMT of LECs. We also studied the relationships between cataract and rat Tpm and human TPM1α/2β expression using lenses from SCR, human cataractous LECs and LECs with lens capsule obtained from intraocular lens (IOL) extraction after non-traumatic post-operative subluxation of IOL. We found the presence of EMT (signalling) in SCR cataractogenesis, which may now be considered a model for exploring various types of deleterious signalling associated with cataractogenesis.

## Materials and methods

### Animals

The present study used 18 adult female albino Sprague–Dawley (SD) rats aged 13 weeks and six female SCRs aged 14 weeks. Cataractous SCR lenses (SCR with cataract) and clear SCR lenses (SCR without cataract) were included. All animal experiments were approved by the Committee of Animal Research at the Kanazawa Medical University, and were conducted in accordance with the National Institutes of Health Guide for the Care and Use of Laboratory Animals and the Institutional Guidelines for Laboratory Animals from the Kanazawa Medical University.

### Surgical procedure

Extracapsular lens extraction (ECLE) was performed in both eyes of all 18 SD rats. Animals were anesthetized with intraperitoneal administration of sodium pentobarbital (Abbott Labs, Abbott Park, IL, USA) at a dose of 40 mg/kg body weight. The surgery was performed by first making a corneal incision with a keratome (Alcon Japan Ltd., Tokyo, Japan). An anterior curvilinear continuous capsulorrhexis was made using capsulorrhexis forceps, followed by lens removal. Animals were killed at either 0 (after the surgery was completed), 7 or 14 days after surgery, *via* a lethal dose of CO_2_. All right eyes were processed for immunohistochemistry studies (*n* = 6 at each time point) and all left eyes for protein blotting.

### Immunohistochemistry

Immunohistochemical procedures were performed on whole eyes from SD rats and SCRs, whole human lenses obtained from the Lions Eye Bank of Nebraska (University of Nebraska Medical Center, Omaha, NE, USA) and five samples from human LECs with capsule obtained from vitrectomy surgery for IOL extraction after non-traumatic post-operative luxation of lens capsules with IOLs. IOLs with human LEC samples were removed from the lens capsules to prepare paraffin blocks and sections. Immunostaining was conducted with a TSA fluorescence system (NEN Life Science Products, Inc., Boston, MA, USA) for human whole lens or DAKO-LSAB Kit for mouse antibody (DAKO, Carpentaria, CA, USA) for human LECs with capsule, as described previously [23, 43, 44]. Tpm1α and 2β were visualized using antimouse Tpm monoclonal antibody (Ab, TM311; Acris Antibodies, Hiddenhausen, Germany), which recognizes Tpm2β isoforms (Tm1; 36 kD) and Tpm1α isoforms (Tm2, 3; 39 kD); antimouse αSMA monoclonal Ab (Sigma-Aldrich, St. Louis, MO, USA) and antirabbit filensin polyclonal antibody (abcam Inc.**,** Cambridge, MA, USA). We controlled for non-specific Ab absorption by adding 5 mg bovine serum albumin (BSA) or the recombinant full-length human Tpm (amino acids 1-284) (abcam Inc.) to a second 5 ml aliquot of the anti-Tpm2β Ab (1:1000 dilution) preparation. Both solutions were incubated overnight at 4°C and then centrifuged at 8000 rpm for 5 min. The supernatants of these two solutions were designated the ‘pre-absorbed anti-Tpm Ab’ and the ‘anti-mouse Tm monoclonal Ab’ preparations. To observe the expression of terminally differentiated lens fibre cells, human tissue samples were immunostained using antirabbit filensin polyclonal Ab (abcam Inc.). To observe the transdifferentiated LECs in EMT, tissues were immunostained using antimouse α-SMA monoclonal Ab (Sigma-Aldrich) in human PCO tissue.

### Western blot analysis

Protein lysates of rat LECs were prepared in ice-cold radioimmune precipitation buffer, and SDS-PAGE and Western blot analysis were performed as described previously [16, 43, 44]. The membranes were probed with antimouse Tpm monoclonal Ab (TM311) and antimouse α-SMA monoclonal Ab (Sigma-Aldrich). Anti-β-actin monoclonal Ab (Sigma-Aldrich) was used to demonstrate that equal amounts of protein were loaded into each lane.

### Human LEC samples obtained from cataract surgery

We prospectively and sequentially examined 90 cataractous eyes in Japanese patients aged 50–85 years who underwent cataract surgery at Kanazawa Medical University between March and October 2007. The type and severity of cataracts were graded and recorded based on a modified version of the LOCS III [45] using six slit-lamp images to grade nuclear colour (NC) and nuclear opalescence (NO), five retroillumination images to grade cortical cataracts (C1-5) and five retroillumination images to grade posterior subcapsular (P) cataracts. Scales on the LOCS III are decimalized and range from 0.1 (completely clear or colourless lens) to 5.9 on the C and P scales (indicating complete opacification of the cortex or posterior capsule) and 6.9 on the NO and NC scales (indicating advanced opacification and brunescence of the nucleus). We classified the 90 samples as being with (+: 14 eyes) or without (−: 76 eyes) ASF. We obtained 90 human LECs with capsule specimens from patients undergoing cataract surgery using a procedure consisting of curvilinear capsulorrhexis.

We obtained approval to conduct this study from the ethics committee of the Kanazawa Medical University (Approval ID: 85). All patients provided informed consent to participate, and patient consent forms were obtained from all participants at the Kanazawa Medical University, Japan. This study adhered to the tenets of the Declaration of Helsinki (2004).

### Real-time reverse transcriptase-polymerase chain reaction (RT-PCR)

Total RNA from the human LECs with capsules and rat LECs was extracted using an RNeasy Micro Kit (Qiagen, Valencia, CA, USA) and following the manufacturer's instructions. To measure the expression of rat Tpm and human Tpm mRNAs, we conducted relative quantification of mRNA using a Prism7300 (Applied Biosystems, FosterCity, CA, USA). PCR amplification was performed with a TaqMan Universal Master Mix and pre-developed rat or human Tpm2β probe mix (Applied Biosystems), which recognize Tm1 isoform, and rat Tpm1α probe mix, which recognizes Tm2, 3 and 5 isoforms. The relative quantity of Tpm1α/2β and Tpm2β mRNA was determined using the comparative Ct method and then normalized using a pre-developed TaqMan ribosomal RNA control reagent VIC probe as an endogenous control (Applied Biosystems). A control human LEC sample for the comparative Ct method was selected from a LEC sample of the cataract score in C1, N1 and P1, obtained from a vitrectomy operation for epiretinal membrane.

### Transfection of Tpm1α and 2β in cultured rat LECs

To overexpress Tpm1α and 2β in LECs, a construct containing a green fluorescent protein (GFP) and full-length human Tpm1α and 2β cDNA were generated using the eukaryotic expression plasmid vector, pReceiver M03 purchased from GeneCopoeia, Inc. (Rockville, MD, USA). Plasmids containing full-length Tpm1α and 2β cDNA were transformed into TOP10F' competent cells (Invitrogen, Carlsbad, CA, USA). These bacterial cells containing plasmid were grown overnight and collected by centrifugation. Plasmids were isolated and used for transfection studies [46, 47]; LECs isolated from SD rats were cultured in Dulbecco's modified eagle medium (DMEM; Sigma-Aldrich) supplemented with 20% foetal bovine serum (FBS; Sigma-Aldrich). Rat LECs at passage 8–10 were used for the study and were transfected with GFP-vector (Vec) cDNA or human Tpm1α or 2β cDNA using Neon™ Transfection System **(**Invitrogen) according to the manufacturer's protocol. pReceiver-M03 empty vector was used as control. To enrich exogenous expression, cells were transfected twice; transfected cells were harvested and cultured onto 60 mm plates. After 72 hrs, these cells were retransfected with the same amount of plasmids (5 μg) encoding Tpm1α or 2β genes. Following 72 hrs of retransfection, cells were harvested and 5 × 10^4^ cells/well were cultured in 12-well plates. The exogenous levels of GFP-linked Tpm1α or GFP-Tpm 2β were assessed using Western blot analysis with antirabbit GFP polyclonal Ab obtained from Professor Etsuko Kiyokawa of Kanazawa Medical University, Japan.

### Statistical methods

The correlation between LOCS III score, ASF+ or −, age and Tpm2β mRNA levels was analysed using one-factor analysis of variance or Pearson's correlation coefficient, with data expressed as mean ± SE. Differences were considered statistically significant at *P* < 0.05.

## Results

### Altered expression of Tpm1α and 2β protein in differentiating LECs of a rodent model of PCO

**Fig. 1:**
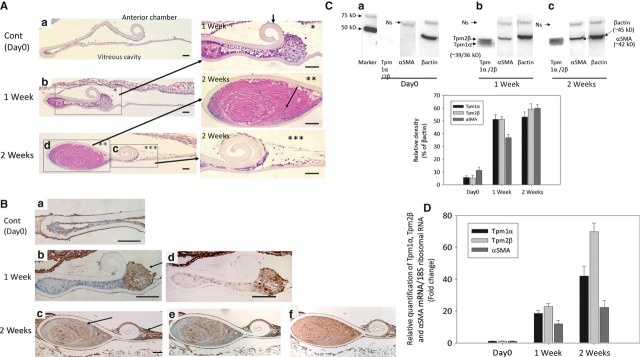
Light microscopy findings (**A**: Haematoxylin and eosin staining) and immunohistochemical images (**B**) of Tpm1α/2β (B-a,b,c), αSMA (B-d,e) and filensin (B-f) in a rat model of PCO after ECLE (the capsular bag at the immediately after ECLE (A-a and B-a), 1 week after ECLE (A-b and B-b) and 2 weeks after ECLE (A-c,d, B-c). Arrows in Figure 1B indicate immunopositive staining of Tpm1α/2β (B). Expression analysis showing levels of protein (**C**) and mRNA (**D**) of Tpm1α and 2 in rat model of PCO after ECLE using Western analysis and qRT-PCR, respectively. (A: left bar = 200 μM, right bar = 100 μM; B: bar = 100 μM). *N* = 6 rats. Ns, a faint protein band (∼70 kD) appeared in lanes immunoblotted with anti-α-SMA or anti-β-actin antibody with approximately the same intensity, suggesting it is non-specific. This band also suggested equal loading because it remains constant, demonstrating that not all proteins are altered in cells.

We performed light microscopic observation to analyse phenotypes and migration patterns of LECs following ECLE. As shown in Figure 1A, immediately after ECLE the capsular bag appeared clean, with LECs present only under the anterior capsule and at the equatorial region (Fig. 1A-a). However, 1 week after ECLE, elongated LECs were displaced interiorly, covering the inner surface of the posterior capsule (Fig. 1A-b). These abnormal changes proved to be progressive, and 2 weeks after ECLE, the posterior capsule was covered with elongated and fibroblastic LECs (Fig. 1A-c), and new lens formation was apparent in the periphery of the capsular bag (Fig. 1A-d).

Given our previous study demonstrating that Tpm1α and 2β are highly expressed in LECs [23], we next sought to define the expression patterns of these two molecules in the rodent model of PCO. Immunohistochemical images of Tpm1α/2β in a rat model of PCO are shown in Figure 1B. In test animals at Day 0 (killed immediately after ECLE), faint staining of Tpm1α/2β protein expression was observed in LECs (Fig. 1B-a). Subsequently, however, the expression pattern of these proteins appeared to be shifted. One week after ECLE, Tpm1α/2β was strongly expressed in elongated LECs (Fig. 1B-b). Interestingly, a similar pattern of α-SMA expression was observed (Fig. 1B-d). Two weeks following ECLE, Tpm and αSMA were strongly expressed in fibroblastic LECs and moderately expressed in the newly formed lens fibres (Fig. 1B-c and B-e). A marker for lens fibre, filensin immunostaining, revealed positive staining of the newly formed lens fibres (Fig. 1B-f).

Next, we examined the expression pattern of Tpm1α/2β protein in samples extracted from lens capsules after ECLE/ASF using Western blot analysis with Ab specific to Tpm1α/2β. At Day 0, expression of Tpm1α/2β protein was relatively low, showing a faint band (Fig. 1C-a). At 1 and 2 weeks after ECLE, however, protein expression of Tpm1α/2β in PCO lens capsules was markedly elevated (Fig. 1C-a). Anti-αSMA Ab was used as a positive control (Fig. 1C-b) and showed αSMA expression only in the PCO lens capsules (Fig. 1C-b). Anti-β-actin Ab, which was used as a loading control, showed that an equivalent amount of protein was loaded onto SDS-PAGE. No significant difference was noted between groups (Fig. 1C-b and-c). However, a faint band (Ns, ∼70 kD) appeared in all lanes immunoblotted with anti-αSMA or anti-β-actin Ab with approximately the same intensity, suggesting it is non-specific. Interestingly, this band reflects equal loading because it remains constant from Day 0 to Week 2, demonstrating that not all proteins were changed. Next, we performed real-time PCR to examine Tm1 isoform from Tpm2β gene and Tm2, and Tm3 and 5 isoforms from Tpm1α genes. Expressions of Tpm1α/2β and αSMA mRNAs were low at Day 0. At 1 and 2 weeks after ECLE, however, mRNA expression of Tm isoforms from Tpm1α/2β genes and αSMA in PCO lens capsules were elevated (Fig. 1D). Taken together, these findings indicate that fibroblastic LECs in patients with PCO expressed Tpm1α/2β and αSMA.

### Tpm1α/2β protein expression in whole lenses and cultured LECs from SCRs with and without cataracts

**Fig. 2:**
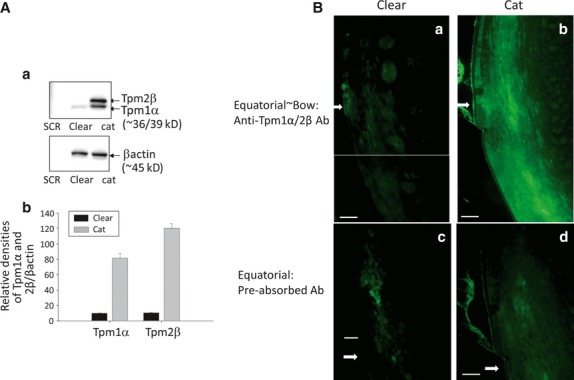
Western blot and immunohistochemical images of Tpm1α/2β in SCR lenses. (**A**) Protein blot shows that the expression of Tpm1α/2β protein was significantly increased in lenses from SCRs with cataract (Cat), compared with the lenses from SCR without cataract (Clear) (Blot:A-a, Relative Density of Blot: A-b). *N* = 6 rats. (**B**) Expression of Tpm1α/2β protein in the equatorial to posterior bow regions of lens from SCR without cataract was low compared with lenses from SCR with cataract. Tissue sections were processed and immunostained with Ab specific to Tpm1α/Tpm 2β. Tpm1α/2β was strongly expressed in cataractous LECs (Cat: SCR with cataract). Tpm1α/2β was localized in the cytoplasm of LECs and surface lens fibres in cataractous lenses (Cat; B-b). In contrast, Tpm1α/2β immunostaining was decreased in non-cataractous clear lenses from SCR (Clear; B-a). A section stained with pre-absorbed Tpm1α/2β Ab served as a control; non-specific staining was observed in the equatorial regions of both SCR without cataract (Clear; B-c) and SCR with cataract (Cat; B-d) lenses. Arrow indicates the bow region of lens. (bar = 100 μM).

To monitor the level of Tpm1α/2β protein in the cultured LECs from SCRs, protein extracts were resolved through SDS-PAGE, and Western blot analysis was conducted using an Ab specific to Tpm1α/2β (TM311). In LECs from SCR without cataract, the Tpm1α/2β band (36/39 kD) was very faint (Fig. 2A-a and A-b; black bar), whereas in cells derived from SCR with cataract, the band was quite strong (Fig. 2A-a and A-b; grey bar). Relative density of Western blot (A-b) revealed that Tpm1α and 2β expression was significantly increased in the latter cells. Immunohistochemical analysis of Tpm1α/2β proteins in lenses from SCR without cataract showed weak staining in the cytoplasm of LECs and lens fibres (Fig. 2B-a). In contrast, intense positive immunostaining in the cytoplasm of LECs and lens fibres was observed in lenses derived from SCR with cataract (Fig. 2B-b).

### Immunohistochemical analysis of Tpm1α/2β localization in human lenses and LECs

**Fig. 3:**
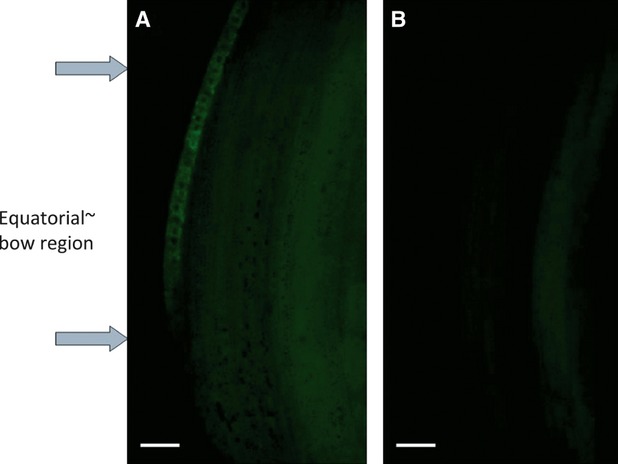
Immunohistochemical images of Tpm1α/2β in human whole lenses. Tpm1α/2β protein was expressed in the cytoplasm of LECs immunostained using anti-Tpm1α/2β Ab (**A**). Specific staining of Tpm1α/2β was not detected in LECs immunostained with pre-absorbed anti-Tpm1α/2β Ab (**B**) (Bar = 80 μM). Arrow indicates the equatorial to the bow region of human lens.

**Fig. 4:**
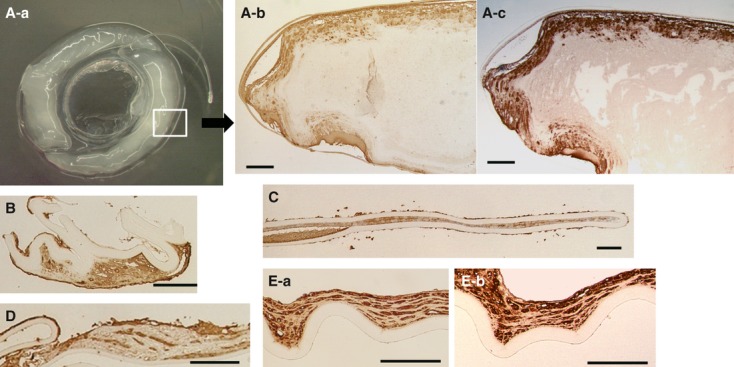
Immunohistochemical images of Tpm1α/2β in human LECs with capsules obtained by vitrectomy surgery from non-traumatic dislocated IOLs covered with lens capsules. The lens capsules with dislocated IOLs were filled with white material generated by the LECs remaining in the peripheral area of the capsular bag (**A**-a). Filensin was immunostained as a marker for lens fibre. Colocalization of Tpm1α/2β (A-b) and filensin (A-c) suggested that Tpm1α/2β was expressed in the newly formed lens fibre in the anterior region of the capsular bag. Tpm1α/2β was expressed in fibroblastic LECs generated from the LECs remaining in the capsular bags (**B**)–(**E**) (Bar = 100 μM). Fibroblastic LECs were immunostained using anti-αSMA Ab, which is known as an EMT marker (E-b) (Bar = 100 μM).

Given our observation of elevated Tpm1α/2β protein expression during EMT of LECs in the rodent model of PCO, we next examined whether these two proteins were expressed in human whole lens and/or LECs obtained from non-traumatic dislocated IOLs covered with a lens capsule. We determined the localization of Tpm1α/2β protein using the anti-Tpm1α/2β monoclonal Ab. In human whole lens samples obtained from the Lions Eye Bank of Nebraska (University of Nebraska Medical Center, Omaha, NE, USA), Tpm1α/2β was stained in the cytoplasm of LECs and was not stained in fibre cells (Fig. 3A). In contrast, specific staining of Tpm1α/2β protein in the cytoplasm of human LECs was not observed in negative control samples stained with pre-absorbed anti-Tpm1α/2β Ab (Fig. 3B). Furthermore, intense staining was observed in human LECs (man, 75 years old) with capsule obtained by vitrectomy surgery from non-traumatic dislocated IOLs at Kanazawa Medical University, which were covered with lens capsules (Fig. 4A-a). Also, strong immunostaining was observed in the terminally differentiated lens fibres at the bow region (Fig. 4A-b), which was also stained with anti-filensin Ab (Fig. 4A-c). Filensin is a lens-specific intermediate filament protein, expressed in the lens fibre [48]. LECs with PCO in four cases (81-year-old woman, Fig. 4B; 72-year-old man, 4C; 52-year-old man, 4D; 85-year-old woman, 4E) were also immunostained using anti-TPM Ab. Intense staining with use of anti-Tpm1α/2β Ab (Fig 4E-a) was observed in fibroblastic and differentiated LECs, which were also immunostained using anti-αSMA Ab (Fig. 4E-b). The intense staining of the human PCO samples suggests that Tpm1α/2β expression may be involved in the EMT process of human LECs, similar to the effects observed in the rat model of PCO.

### Relationship between expression of Tpm2β mRNA, severity of nuclear cataract and cataracts with anterior subcapsular fibrosis (ASF) in human LECs

**Fig. 5:**
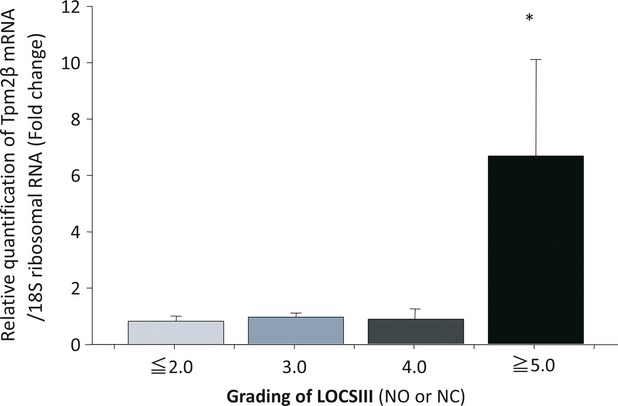
Severity of nuclear cataracts and relative expression of Tpm2β mRNA in human LECs. Expression of Tpm2β was significantly higher in nuclear cataracts in the samples with cataracts graded ≥NO5 or ≥NC5. Data are shown as mean ± SE (Pearson's correlation coefficient; 0.445, *P* < 0.0001).

We used Tpm2β (Tm1 isoform) to determine whether expression levels of Tpm are associated with human cataract, as this protein was more highly expressed than Tpm1α in rat PCO (Fig. 2) and *Prdx6*^−/−^ mouse LECs [23]. Figure 5 correlates the severity of nuclear cataracts with the relative expression of Tpm2β mRNA in human LECs. Expression of Tpm2β was significantly higher in the sample group with severe nuclear cataract (≥Grade 5.0) than it was in the groups with lower grades of nuclear cataract (≤Grades 2.0, 3.0, 4.0) (Pearson's correlation coefficient; 0.445, *P < 0.0001). Grading was based on a modified version of the lens opacities classification system (version III; LOCS III) [45]. However, no significant associations were noted between severity of cortical or posterior subcapsular cataracts or age and Tpm2β mRNA levels.

To identify the correlation between expression of Tpm2β mRNA and the presence of ASF with cataracts in human LECs, we isolated RNA from two groups of samples—one with ASF and one without—and conducted real-time PCR. Table 1 shows expression levels of Tpm2β in the two groups. Expression of Tpm2β in LOCS III Grades 3 and ≥5 (NO or NC) with ASF was significantly higher than in those without ASF (*P < 0.020 and *P < 0.046, respectively). Expression of Tpm2β mRNA in all eyes was significantly higher in eyes with ASF (*P < 0.0002) than in those without ASF, suggesting a possible role for Tpm2β proteins in ASF.

**Table 1:** Relative quantification of Tpm2β mRNA/18S ribosomal RNA (Fold change) and grading of nuclear cataract (LOCS III: NO or NC) with or without ASF

LOCS Grade	Asf (−)	Asf (+)	*P*-value (− *versus* +)
≤2	0.753 ± 0.163 (*n* = 24)	2.09 (*n* = 1)	–
3	0.8433 ± 0.1113 (*n* = 32)	1.4743 ± 0.292 (*n* = 9)	<0.020^*^
4	0.8233 ± 0.1613 (*n* = 15)	– (*n* = 0)	–
≥5	1.6263 ± 0.5073 (*n* = 5)	13.164 ± 5.361 (*n* = 4)	<0.046^*^
All	0.8637 ± 0.0845 (*n* = 76)	4.8579 ± 2.0132 (*n* = 14)	<0.0002^*^

Values are mean ± SE. *n*: eyes; –: indefinite. ^*^P < 0.05.

### Effect of Tpm1α and/or Tpm 2β overexpression in cultured rat LECs

**Fig. 6:**
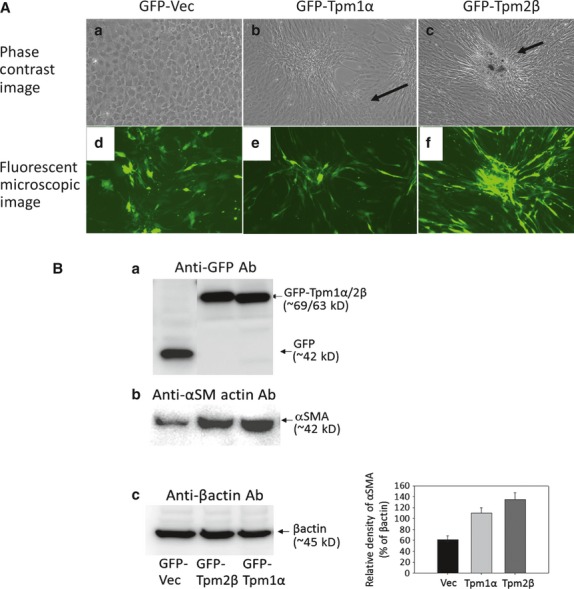
Overexpression of Tpm1α and 2β induced the differentiation of cultured rat LECs. (**A**) Microscopic images of human Tpm1α- and 2β-transfected rat LECs. In rat LECs transfected with GFP-Vec, cell morphology was normal and not elongated (Phase contrast image: A-a, Fluorescent microscopic image: A-d). In rat LECs overexpressing Tpm1α, the cells were elongated and showed fibroblastic changes (A-b, e: arrow = fibroblastic changes). Overexpression of Tpm2β induced the fibroblastic changes and the formation of lentoid body in rat LECs (green fluorescence indicating overexpressing cells,) (A-c, f). The cells showed fibre-like structure, clustered and formed lentoid body (A-c: arrow = lentoid body). (**B**) Protein blot of Tpm1α- and 2β-transfected rat LECs using anti-GFP, Ab (a), anti-αSMA Ab (b) and anti-βactin Ab (c). Rat LECs overexpressing Tpm1α and 2β displayed the up-regulation of α-SMA (B-b, c). Aa–c, phase contrast images; Ad–f, fluorescent microscopic images. *N* = 4 trials.

To determine whether rat LECs overexpressing human Tpm1α or Tpm2β develop abnormal phenotypes and display elevated expression of α = SMA, we compared rat LECs transfected with Tpm1α or Tpm2β linked to eGFP to those transfected with empty vector. Data revealed that overexpression of Tpm1α and 2β induced elongation and fibroblastic changes in LECs (Fig 6A-b,c,e,f) overexpressing Tpm1α or 2β (green colour). Also, formation of lentoid body was observed in LECs overexpressing Tpm2β (Fig. 6A-c,f). Cells overexpressing Tpm1α or 2β recombinant protein showed fibre-like structure (f, green colour), indicating development of abnormal phenotypes.

Next, we performed Western analysis to examine whether these cells displayed elevated expression of α-SMA. Transfected LECs expressed GFP-Tpm1α or Tpm2β and GFP-Vector, showing that the overexpression of each protein was properly induced (Fig. 6B-a). Expression of α-SMA was up-regulated in cells overexpressing Tpm1α and/or Tpm2β (Fig. 6B-b, c). In non-transfected rat LECs, expression of α-SMA protein was not detected (data not shown). In contrast, cells overexpressed with empty vector did not show any adverse changes, suggesting that the adverse changes in cells were Tpm mediated.

## Discussion

We assessed the expression of Tpm, specifically high-molecular-weight Tm isoforms from Tpm1α and 2β genes, in a rodent model of PCO and in LECs obtained from cataractous human patients of various ages. We showed that expression of Tpm1α/2β was minimal in rat LECs. However, we found that expression of Tpm1α/2β increased during EMT and demonstrated that selective elevation of Tpm1α/2β in rat LECs compared with controls was correlated with fibrosis observed in PCO. Expression of α-SMA, a marker of differentiation and EMT of LECs, was colocalized with Tpm1α/2β in rodent PCO. Data from the present study suggest that the expression of Tpm1α/2β may be associated with progression of PCO. Several previous studies have examined the role of modulated expression of Tpm proteins in EMT and these proteins' roles in initiation and progression of a range of diseases [34, 49, 50]. In the present work, we report for the first time that expression of Tpm1α/2β was induced/elevated and transdifferentiated into multilayered, spindle-shaped LECs in a rat model of PCO, in human cataracts with ASF and in human differentiated LECs in a dislocated lens capsule. No studies to date have focused on the role of Tm isoforms in LECs, as rat Tpm and human Tpm1α/2β expression are extremely low in human and rat LECs. Previously, we noticed similar changes in expression levels of Tpm1α/2β in *Prdx6*^−/−^ mouse LECs [23]. Expression of Tpm1α/2β in these LECs was surprisingly high compared with wild-type LECs (*Prdx6*^+/+^ >120-fold higher) and was localized in stress fibres. *Prdx6*^−/−^ LECs are more sensitive to oxidative stress than wild-type LECs, and changes in *Prdx6*^−/−^ LECs in the present study involved increased ROS levels [15]. ROS activate TGF-β, which is an inducer of oxidative stress and cataract development [15, 51, 52]. Expression of Prdx6 was reduced in lenses from SCR with cataract. We believe that elevated generation of ROS due to reduced expression of antioxidants can contribute to alteration of redox homeostasis, which subsequently compromises cellular ability to respond to additional insults, as seen in lenses from SCR with cataract [19]. Cataractogenesis in SCR might be associated with ROS-mediated activation of TGFβ1 that in turn leads to EMT. Moreover, work by our group and others has shown the molecular mechanism whereby ROS-induced TGFβ1 up-regulates αSMA gene expression [15, 23], which in turn leads to EMT and cataractogenesis [8, 53–55].

Recent evidence reveals that gene mutation or mutant proteins also induce oxidative stress. In the SCR, cataractogenesis is caused by mutations in the lanosterol synthase gene [38]. Cataractogenesis in the SCR shows a spectrum of biochemical and morphological changes, which resemble those seen in cataractogenesis or pathogenesis induced by oxidative stress and growth factor TGFβ [7, 8, 15, 52, 56]. Using the SCR as an experimental model, we have demonstrated that LECs from these rats contain elevated levels of ROS and bioactive TGF-β1 with overmodulation of TGF-β1-inducible genes such as αSMA and βig-h3, genes which have been implicated in the pathophysiology of cataractogenesis [12, 13, 20, 51]. Several TGF-β target genes, including α- and β-Tpms, α-actinin1 and calponin2-encoding actin-binding proteins, have been implicated in the assembly of stress fibres [33, 34]. Of these, Tpms in particular have been shown to play a crucial role in stabilizing actin filaments [35]. TGF-β specifically up-regulates expression of α- and β-Tpm genes, but has no effect on regulation of Tpm3 and Tpm4 genes, which encode low-molecular-weight Tpms [33, 34]. In addition, our research revealed up-regulation of Tpm1α/2β in differentiating LECs, suggesting the involvement of TGF-β-induced deleterious signalling in the induction of Tpm1α/2β. We hypothesize that activation of TGF-β is induced by both surgical stress and ROS during cataract surgery, which consequently induces EMT by up-regulating Tpm1α/2β genes, leading to PCO. However, further in-depth studies will be required to fully clarify the underlying mechanism of Tpm1α/2β's involvement in this process.

In the immunohistochemical study, we found that expression of Tpm1α/2β was increased in human LECs obtained from dislocated lens capsule with IOLs following cataract surgery. Previous *in vitro* capsular bag experiments have established the presence of TGF-β-induced abnormal changes in LECs [57]. Although members of the TGF-β superfamily have been implicated in lens fibre differentiation, the inappropriate TGF-β signalling in anterior LECs results in an EMT that bears morphological and molecular resemblance to certain human cataracts including ASF and PCO [5, 10]. Induction and involvement of Tpm isoforms and stress fibres has been suggested to play a major role in TGF-β control of cell migration and architecture and is necessary for TGF-β-mediated formation of stress fibres [33]. For example, EMT and stress fibre formation of LECs have been observed in anterior subcapsular fibrosis of human cataracts and PCO following cataract surgery [40–42]; these changes may actually be effected by induction of Tpm1α/2β by TGF-β. Furthermore, we found that overexpression of human Tpm1α/2β induced the fibroblastic changes, lentoid body formation and up-regulation of α-SMA expression in rat LECs. However, rat LECs transfected with empty vector also expressed α-SMA, but the expression level of α-SMA was significantly elevated in rat LECs overexpressing Tpms. We believe that expression in empty vector-transfected rat LECs may be related to *in vitro* cell culture shock. As a whole, our work suggests that Tpm1α or Tpm2β promotes the EMT of LECs. Regardless of the pathophysiological importance of Tpm isoforms, we believe that the distinct pattern of Tpm2β expression observed in this study may function as a clinical marker of LEC differentiation, posterior capsule opacification or ASF. Furthermore, we hope that the Tpm2β expression pattern data will provide insight into developing inhibitors of Tpm1α/2β, subsequently suppressing EMT of LECs as well as PCO and ASF.
